# Do musicians learn a fine sequential hand motor skill differently than non-musicians?

**DOI:** 10.1371/journal.pone.0207449

**Published:** 2018-11-21

**Authors:** Jagna Sobierajewicz, Ryszard Naskręcki, Wojciech Jaśkowski, Rob H. J. Van der Lubbe

**Affiliations:** 1 Vision and Neuroscience Laboratory, NanoBioMedical Centre, Adam Mickiewicz University, Poznan, Poland; 2 Laboratory of Vision Science and Optometry, Faculty of Physics, Adam Mickiewicz University, Poznan, Poland; 3 Institute of Computing Science, Poznan University of Technology, Poznan, Poland; 4 Cognitive Psychology and Ergonomics, University of Twente, Enschede, The Netherlands; Universita degli Studi di Verona, ITALY

## Abstract

Do professional musicians learn a fine sequential hand motor skill more efficiently than non-musicians? Is this also the case when they perform motor imagery, which implies that they only mentally simulate these movements? Musicians and non-musicians performed a Go/NoGo discrete sequence production (DSP) task, which allows to separate sequence-specific from a-specific learning effects. In this task five stimuli, to be memorized during a preparation interval, signaled a response sequence. In a practice phase, different response sequences had to be either executed, imagined, or inhibited, which was indicated by different response cues. In a test phase, responses were required to familiar (previously executed, imagined, or inhibited) and unfamiliar sequences. In both phases, response times and response accuracy were measured while the electroencephalogram (EEG) was only registered during the practice phase to compare activity between motor imagery, motor execution, and motor inhibition for both groups. Results in the practice phase revealed that musicians learned the response sequences faster and more accurately than non-musicians although no difference in initiation time was found. EEG analyses revealed similar lateralized activity during learning a motor skill for both groups. Our results from the test phase showed better sequence-a-specific learning effects (i.e., faster response times and increased accuracy) for musicians than for non-musicians. Moreover, we revealed that non-musicians benefit more from physical execution while learning a required motor sequence, whereas sequence-specific learning effects due to learning with motor imagery were very similar for musicians and non-musicians.

## Introduction

Playing the piano is a highly complex motor task that requires a lot of practice. To play a piece of music one regularly has to coordinate a sequence of finger movements between two hands at different tempi. For example, for one measure, the left hand may need the simultaneous and subsequent key presses of two keys at a constant rhythm of three per measure, while the right hand requires six different single key presses at a constant rhythm within the same measure. The common way in which people learn to play a piece of music is by repeating it over and over again. However, it is also known that professional musicians use additional strategies like mental imagery (i.e., auditory and/or motor imagery) to improve their performance [1]. As musicians have to train fine motor skills significantly more often than novices, learning related motor skills may be easier for musicians than for non-musicians. The learning of a motor skill is thought to involve both a motor and a more cognitive level [2,3,4], and recent research suggests that motor imagery may only be related to a cognitive level [5]. The potential benefit for musicians may thus be related to this cognitive and/or motor level, and one might propose that musicians additionally benefit from motor imagery. We hypothezised that this increased ability to perform motor imagery by musicians might also be visible in electrophysiological measures that reflect the involvement of relevant brain areas.

Learning to execute a motor skill can be examined with the so-called discrete sequence production (DSP) task, while the Go/NoGo variant of this task [6] seems a perfect tool to study learning by motor imagery, and compare this with learning by motor execution (see [7]). The standard DSP task cannot be used to study motor imagery as in that task responses are required to trigger the next stimulus. In the Go/NoGo DSP task, a visually presented stimulus sequence has first to be memorized. Subsequently, after a Go signal the sequence should be either executed by pressing the relevant buttons, or the sequence has to be mentally imagined, while after a NoGo signal the action should be simply withheld (or inhibited). A major advantage of this task is the possibility to separate particular stages of brain activity during the acquisition of motor skills, i.e., encoding the stimuli, memorizing their sequence, preparing the responses, and finally executing the responses (either mentally or physically). During a practice phase different sequences of equal complexity are executed, imagined, and inhibited. Sequence-specific learning effects can subsequently be assessed by executing the previously executed, imagined and inhibited sequences in a test phase and comparing their performance with unfamiliar sequences. The difference between previously executed and unfamiliar sequences reflects the sequence learning effect of response execution, the difference between previously imagined and unfamiliar sequences reflects the sequence learning effect of motor imagery, and the difference between previously withheld and unfamiliar sequences reflects the sequence learning effect of motor inhibition. Sobierajewicz et al. (2017a) demonstrated with this paradigm that motor execution and motor imagery during the practice phase both induce sequence-specific learning effects. Furthermore, event-related lateralizations (ERLs) derived from electroencephalographic (EEG) recordings revealed increased contralateral negativity above motor areas during both motor execution and motor imagery trials, while no such effect was observed in a control condition [7]. The latter results suggest that relevant motor areas are activated during both motor execution and motor imagery, which corroborates with several earlier findings [8,9]. In another study, it was examined whether learning effects of motor execution and motor imagery were muscle-specific or not [5]. This was done by varying the execution mode as sequences were either learned with four fingers or only with the index fingers of both hands. Results revealed that sequence-specific learning effects were not dependent on the execution mode during the practice phase, which suggests that these effects are not muscle-specific. These findings can be understood within the framework for sequential motor behavior as proposed by Verwey et al. (2015),[4].

Verwey et al. (2015) proposed that two different representational levels may be involved while learning to produce a sequence of movements. A cognitive level is thought to relate to spatio-temporal aspects of the movement sequence which already develops with limited practice. A second motoric level is thought to relate to the involved muscles or muscle groups [10], but representations at this level develop only after extended practice [11]. Thus, representations at a cognitive level develop faster (especially in the initial phase of the learning of a motor sequence) than motor or muscle-specific representations. The possible difference between musicians and non-musicians might in principle involve both types of representations. Thus, musicians might be better able to learn new spatio-temporal patterns which may show up in the benefits of learning by motor execution and motor imagery. Alternatively, musicians might already involve motor-specific representations at an earlier stage. In that case, the benefit would be most pronounced when learning by motor execution. Another possibility is that the potential benefit is related to a general increase in fine motor control that is not sequence-specific. In that case, the benefit for musicians would also be present for unfamiliar sequences.

Several researchers showed that differences in behavioral performance between musicians and non-musicians may be related to specific brain structures [12, 13, 14, 15, 16]. Most of the studies investigating music performance or music improvisation focused on the role of frontal brain regions [16, 17, 18, 19, 20]. Moreover, it has been revealed that the multiple-demand system (consisting of several areas in the prefrontal and parietal regions) is involved in many complex activities among musicians[21]. For example, it has been shown that activation of the multi-demand system is related with response selection, working memory, task novelty, and attentional control for goal-oriented behavior [21, 22, 23, 24]. Based on fMRI (functional magnetic resonance imaging) results, it has been proposed that highly trained individuals (as compared to novices) are characterized by a decrease in the overall volume of brain activation (e.g., the fronto-parietal network) while they display an increased activation of brain areas relevant for executing the task (e.g., the primary motor cortex), [12, 25]. In line with this proposal, an fMRI study by Lotze et al. (2003) revealed that professional musicians display more focused activation patterns during musical performance than amateurs. A recent EEG study of Zhao et al. (2017) additionally showed a reduction of the mismatch negativity (MMN) for musicians relative to non-musicians while passively listening to strong and weak tones. The latter results may indicate that musicians use less cognitive resources, which would be indicative of increased processing efficiency [26]. However, EEG results from another study of Bianco et al. (2017) suggested that attentional control, indexed with a prefrontal negativity and the P3 component, was enlarged for musicians (i.e., drummers) as compared with non-drummers while performing a visuo-motor discriminative response task [27]. Bianco et al. (2017) explained their results by a long-term neural adaptation mechanism and increased visuo-spatial abilities for drummers [27]. Based on the previous fMRI results [12, 25] showing increased activation of motor areas, we hypothesized that electroencephalographic activity above cortical motor areas while executing a required motor sequence may also be more pronounced for professional pianists as opposed to non-musicians. A way to examine this possibility is to derive ERLs from the EEG. ERLs are highly specific for motor-related processes as activity that is unrelated to the relevant side is subtracted out [7, 9, 28].

The previous section indicates that musicians may learn a motor skill more easily than non-musicians, which may be related to the extra involvement of motor areas. The question remains open whether this also applies to the learning of a motor skill with motor imagery, which implies that relevant movements are mentally simulated without any overt action [29]. Most of the prior studies that aimed to investigate motor learning with motor imagery showed that training with motor imagery can significantly promote the learning of a motor skill, however, in that case the training needs to be very intensive [30, 31]. For example, in the study of Pascual-Leone et al. (1995) participants practiced for two hours per day for a duration of five days, while in the study of Jackson et al. (2003) participants mentally practiced 1500 sequences in each of five training periods. Bernardi, De Buglio, Trimarchi, Chielli, and Bricolo (2013) questioned whether mental practice may optimize movement timing by employing expert pianists who performed difficult music sequences either with mental practice or with physical practice. Changes in performance were observed in movement velocity, timing and coordination. Improved performance was observed after mental practice, although better results were obtained after physical practice [32]. In the study of Brown and Palmer (2013) pianists’ pitch accuracy was measured to examine how auditory and motor imagery abilities affect the learning of novel melodies and recall those melodies. Pianists learned melodies either by performing without sound (motor learning) or by listening without performing (auditory learning). Although results revealed that pitch accuracy was higher after auditory learning than after motor learning, both auditory and motor imagery skills improved pitch accuracy. The above-mentioned studies confirm the benefit of motor imagery on timing and accuracy during learning a motor skill. Given the fact that motor imagery is beneficial for motor skill learning, and it has been revealed that musicians are better in the acquisition of a motor skill than novices, it may be proposed that learning a sequential fine motor skill with motor imagery is more beneficial for musicians than for non-musicians.

In the current study, we first questioned whether learning a fine motor skill with motor execution is more effective for professional musicians than for non-musicians. The potential benefit for musicians may be related to the processing of a motor sequence at both a cognitive and a motor level due to long-term practice and expertise, while for non-musicians a motor sequence may only be reinforced at a cognitive level. We predicted that during the practice phase musicians would learn all motor sequences more efficiently than non-musicians. This would lead to better motor performance (faster and more accurately) in the test phase, indicating a-specific learning effects. Secondly, we hypothesized that due to the increased ability to perform motor imagery, musicians may benefit more from motor imagery than non-musicians while learning a motor skill (i.e., sequences that were learned with motor imagery may be executed faster and more accurately by musicians than by non-musicians, indicating sequence-specific learning effects). To improve our understanding of performance differences between musicians and non-musicians, we examined brain activity during motor execution, motor imagery, and motor inhibition for musicians and non-musicians in the practice phase (i.e., when executing, imaging, and inhibiting particular sequences) by comparing ERLs above cortical motor areas. We expected that the electrophysiological activity is more pronounced for professional pianists as opposed to non-musicians [12,15].

## Methods

### Participants

A sample of 24 healthy volunteers (4 males, 20 females) aged between 21 and 29 (M_age_ = 24.5, SD 2.41) took part in the experiment. All of them reported to have no history of mental or neurological disorders. Musicians (either students of music or graduated musicians) (n = 12, M_age_ = 24.67, SD 1.56, 3 males, 9 females) were recruited mainly from the Ignacy Jan Paderewski Academy of Music in Poznań. The average reported time of daily practice with the piano amounted to 2–3 h (SD 0.71). Musical training started on average at the age of 10 years (SD 3.9). Non-musicians (n = 12, 1 male, 11, females) were recruited mainly from the Adam Mickiewicz University (M_age_ = 24.75, SD 2.9). They reported not having received any formal music education and never learned to play a musical instrument. All participants were requested to complete Annett`s Handedness Inventory [33]. Ten of the musicians were assessed to be right-handed, and two of them were left-handed, while eleven of the non-musicians were assessed to be right-handed, and only one of them was left-handed. All participants gave their written consent before the start of the experiment. Prior ethical approval was granted by the local ethics committee at the Adam Mickiewicz University. The study was performed in accordance with the Declaration of Helsinki.

### Stimuli and task

**Fig 1:**
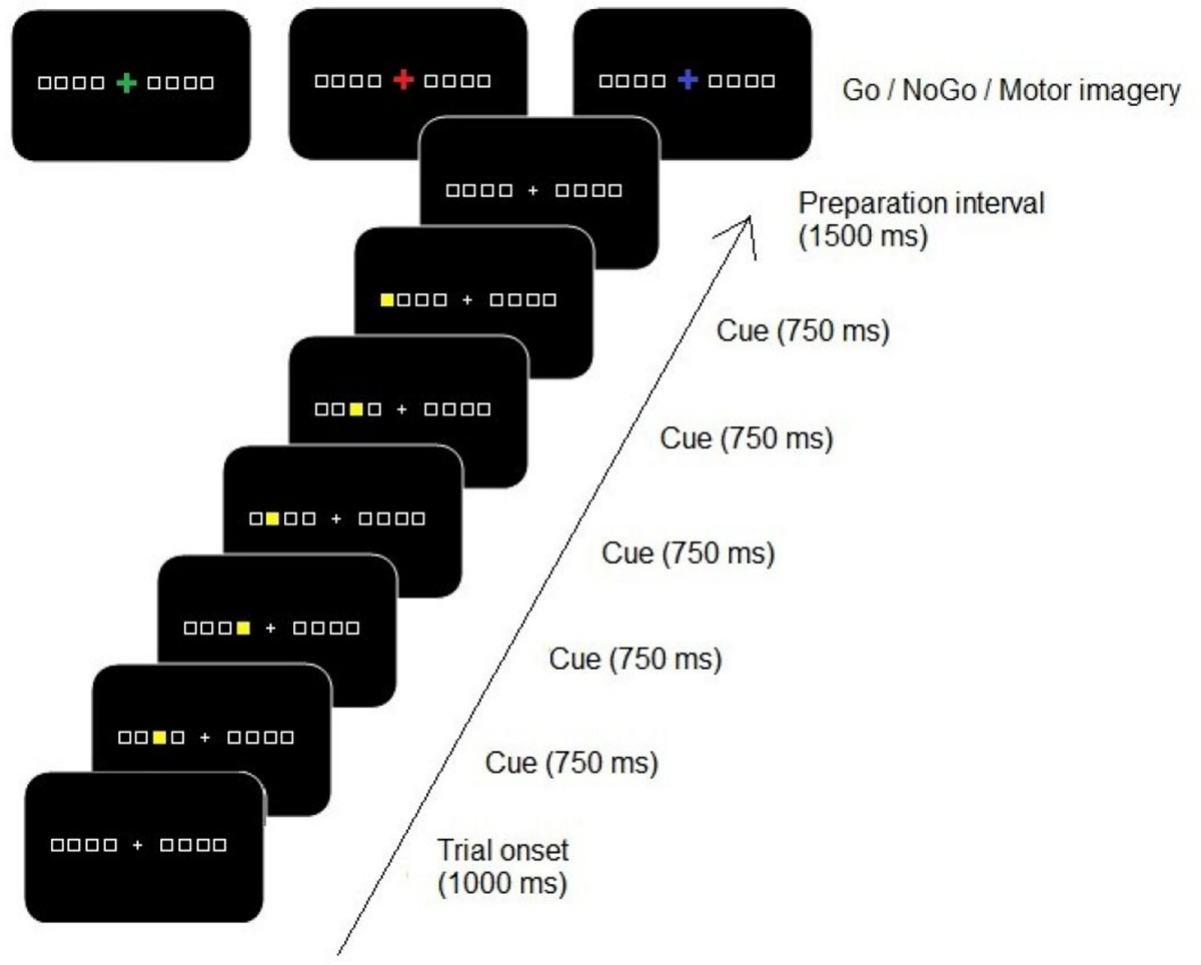
An overview of the sequence of events in the Go/NoGo DSP task. Three possible informative cues were presented after the preparation interval: a green cross implied that the sequence had to be executed (Go signal), a blue cross indicated that execution of the sequence had to be mentally imagined (Go signal), while a red cross indicated that the sequence had to be inhibited (NoGo signal). Participants were instructed to press or imagine pressing the required buttons only after a Go signal, while after a NoGo signal any reaction (also imagined) should be withheld.

An overview of the stimulus sequence on a trial is presented in Fig 1. A sequence of five visual stimuli was displayed on a CRT monitor with a display frequency of 60 Hz. A trial started with a gray fixation cross (1.3°) presented in the center of the screen between eight horizontally aligned squares (2.5°), four on the left and four on the right side of the fixation cross. The squares were black with a gray border, which were presented on a black background. Each square was assigned to a button on the keyboard (a, s, d, f keys and the;, l, k, j keys). The alignment of the eight stimulus squares had a total visual angle of 26.5°. Each trial started with a beep of 300 Hz for 300ms. After a time interval of 1000 ms one of the squares was filled yellow for 750 ms, a second square was filled, etc., until a fifth square was filled. The stimulus sequence was presented to either the right or the left side of the fixation cross. After a preparation interval of 1500 ms relative to the offset of the last stimulus, the Go/NoGo stimulus (a fixation cross) was presented in one of three possible colors in the practice phase. In the case of a green cross, the cued response sequence had to be executed (a Go signal). After a blue cross, the response sequence had to be mentally imagined (a Go signal)—participants should imagine to execute the five spatially corresponding key presses in the same order as the stimulus sequence. In the case of a red cross, the sequence had to be withheld. Thus, only after a Go signal, the volunteer had to reproduce the sequence by pressing or imagining to press corresponding buttons on the keyboard, while after a NoGo signal the action should simply be withheld. In the test phase, only a green cross was presented as all sequences (i.e., previously executed, imagined and withheld) had to be physically executed. All participants were requested to keep their eyes directed on the fixation cross during the presentation of the sequence, and during execution of the required task.

### Procedure

Participants were requested to sit relaxed and comfortably in a dimly lit room. At the start of the experiment, all participants placed their little finger, ring finger, middle finger, and index finger of the left hand on the *a*, *s*, *d*, *f* keys, and their little finger, ring finger, middle finger and index finger of the right hand on the;, *l*, *k*, *j* keys of a computer QWERTY keyboard. The distance from the computer screen was fixed at 70 cm for each individual. The experiment was conducted on a single day. At the start of the experiment each participant received instructions about the details of the experiment. Participants performed five training blocks (32 sequences had to be executed, 32 sequences had to be imagined, and 32 sequences had to be withheld in each block, the same number of repetitions for the right and for the left hand was used), and one final test block (32 sequences executed before; 32 sequences imagined before but now executed; 32 sequences withheld before but now executed, and 32 new sequences that had to be executed). In the Appendix the different sequences are shown which were used in order to eliminate finger-specific effects. We used six different structures (12432, 13423, 14213, 13241, 14312, and 21431) with four different versions of sequences per structure, which were counterbalanced across participants and across fingers.

In the practice phase, sequences had to be executed, imagined, or withheld. In the case of motor imagery, participants were instructed to use a first person perspective (i.e., to imagine the sensation of executing a sequence). To avoid the use of visual imagery, an example of visual and motor imagery was given to all participants (“imagine yourself walking on a street–you can see yourself walking”/ “imagine as if you are walking–you imagine your movements during walking”, respectively). Moreover, participants were asked to imagine only a movement and not a sequence of numbers, symbols, or sounds.

Halfway each block and after each block, a pause was provided in which participants were informed on their mean reaction times and error percentages. Feedback about incorrect responses was given after executing a sequence but only when a participant pressed a button before the Go/NoGo signal or when a false button press was made. Participants were asked to execute or imagine the required sequence as fast and as accurately as possible.

### Behavioral parameters

Response time (RT) was defined as the time between the onset of the Go signal and pressing the first key, and as the time between two consecutive key presses within a sequence [6, 34]. For the practice phase, we tested mean RTs by performing an analysis of variance (ANOVA) with repeated measures with Group (2), Block (4), and Key (5, the number of keys to be pressed within a sequence) as factors. For the test phase, an ANOVA with repeated measures was also applied with Group (2), Type of Sequence (4, familiar executed, familiar imagined, familiar inhibited, and unfamiliar), and Key (5) as factors. Error analyses were performed on arcsin transformed data to stabilize variances [35]. A repeated measures analysis of variance (ANOVA) was performed both for the practice phase with Group (2), and Block (4) as factors, and for the test phase with Group (2), and Type of Sequence (4) as factors. In order to test the prediction that musicians are better in the acquisition of a motor skill (either with motor execution or motor imagery), one-tailed *t*-tests were used.

### EEG parameters and data processing

EEG, EOG, EMG data, and markers signaling the onset of employed stimuli and the specific button presses were registered with Vision Recorder software (Brain Products–version 2.0.3). The EEG was recorded using an ActiCap (BrainProducts, GmbH) with 64 active channels, which were placed on standard locations according to the extended International 10–20 system [36]. A built-in average reference of the amplifier was used. Although a reference electrode standardization technique (REST) may be considered a very useful method in EEG recordings [37, 38, 39, 40], we used an average reference as for contralateral-ipsilateral difference potentials reference-related differences are subtracted out. Electrode impedances were kept below 5kΩ. To monitor ocular artifacts, the vertical and horizontal electrooculogram (vEOG and hEOG) was recorded using bipolar electrodes located above and below the right eye and on the left and right outer canthi, respectively.

Offline analyses were performed with Brain Vision Analyzer software (version 2.0.4). First, EEG data from the practice phase was low-pass filtered (30 Hz). Next, we selected the segments from -2500 ms to 4000 ms relative to the Go/NoGo signal. A baseline was set from -100 ms to 0 ms. We focused on the 1000 ms time interval after the Go/NoGo signal, as we wanted to contrast motor execution with motor imagery and motor inhibition. Trials with major artifacts were excluded from further analysis (maximum allowed voltage step: 100 μV/ms, minimum/maximum allowed amplitude: -/+150 μV, lowest allowed activity within 50 ms intervals: 0.1 μV). A semiautomatic Independent Component Analysis (ICA) was used in order to remove residual activity due to horizontal or vertical eye movements from the EEG (averaged number of removed components: 3.5).

### ERL measures

Contralateral-ipsilateral difference potentials (ERLs) were computed for the practice phase as we wanted to investigate EEG patterns for musicians and non-musicians while learning a motor skill. ERLs were derived from the ERPs, which were determined for each type of task and also per hand. ERLs are based on a double subtraction procedure performed on ERPs computed for left and right hand trials, which extracts activity that is specific to the relevant side: ERL = ((LH(contra-ipsi) + RH(contra-ipsi)))/2.

ERLs were determined for all symmetrical electrode pairs, but on the basis of earlier results statistical analyses were restricted to two electrode pairs: C3/C4, CP3/CP4, [7, 41]. ERLs were analyzed in 40 ms intervals from 0 until 1000 ms after the Go/NoGo signal. Repeated measures ANOVAs were performed for two electrode pairs with the factors Group (2), Time Window (25), and Type of Sequence (3). With 25 time windows from 0 to 1000 ms, the critical *p* value for two successive time windows was estimated at 0.03 (*p*_crit_ < √(0.05/((time windows—1) × electrodes)) < 0.03), [42]. This procedure was applied to reduce the possibility of a Type I statistical error [43].

### EMG

In order to control whether participants flexed their muscles only in the case of motor execution, we measured EMG activity in the practice phase. It was measured bipolarly by attaching EMG electrodes on the musculus flexor digitorum superficalis and on the processus styloideus ulnae of the right and left hand.

To analyze EMG activity, a band-pass filter from 20 to 50 Hz was applied. Next, a wavelet analysis was performed to determine the extent of motor activation in the required motor task. The threshold for a movement was set at 80–120 μV depending on the resting level of the individual participant. A complex Morlet wavelet was chosen (c = 5) with the lower and upper boundaries for the extracted layer set at 20 and 50Hz. To perform the analyses, we choose the time window from the Go/NoGo signal until 5000 ms as it was time to execute or imagine the sequence. The EMG signal was analyzed with the following factors: Group (2), EMG-channel (right relevant hand, left relevant hand, 2), Block (4), and Type of Sequence (3, motor execution, motor imagery, and motor inhibition).

## Results

All statistical analyses were performed with SPSS (IBM Statistics SPSS 22). *P* < 0.05 was chosen as the level of significance for the behavioral and EMG results. Greenhouse–Geisser ɛ correction was applied whenever appropriate.

### Behavioral results

#### The practice phase

**Fig 2:**
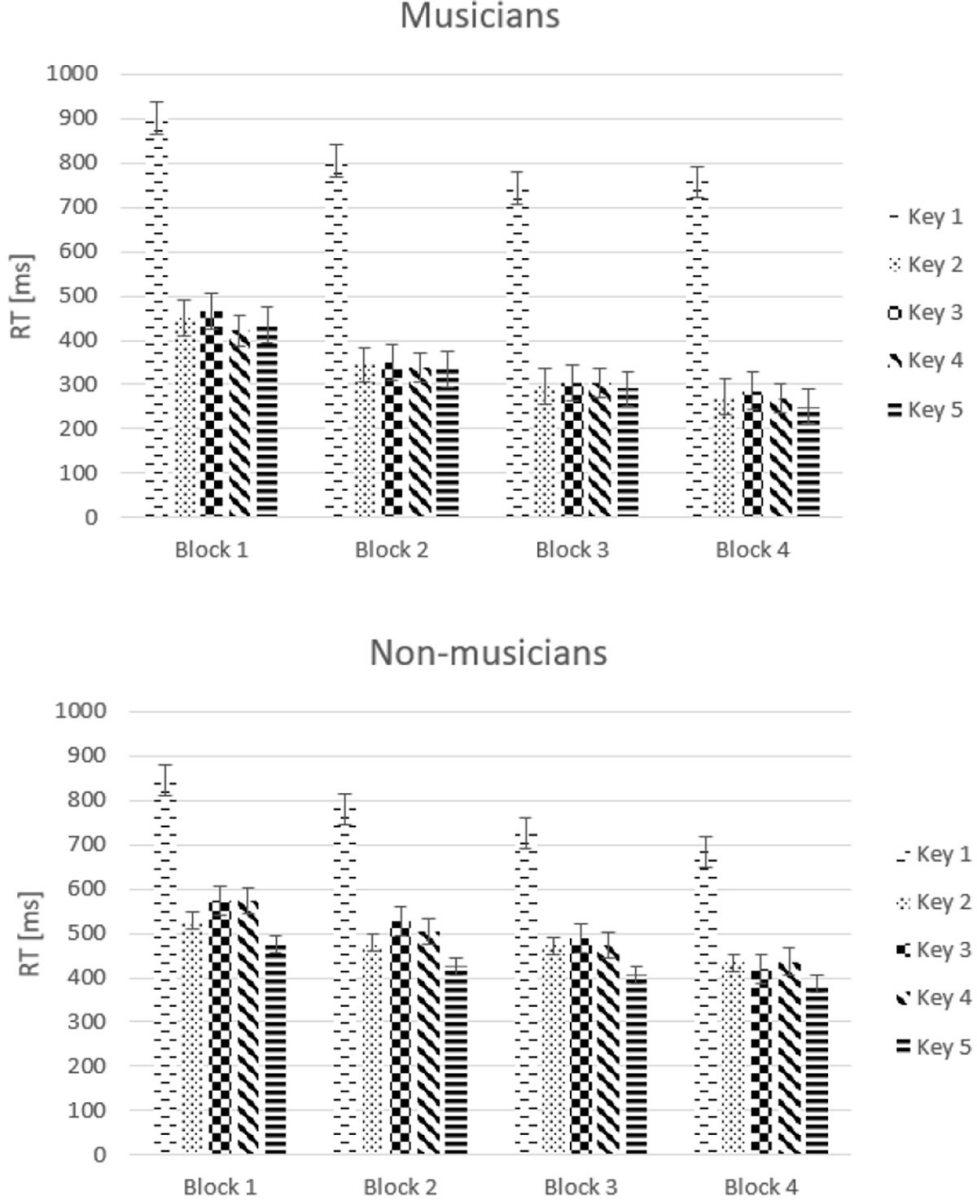
Mean response times (RTs) in milliseconds (ms) for each key press (Key 1–5) as a function of Block for both groups. *Error bars* represent standard errors.

Fig 2 gives an overview of mean RT results from the practice phase for both groups as a function of Key. The results revealed a trend to a significant difference in mean RTs between musicians and non-musicians (mean RTs for keys 1 to 5 for musicians were 802, 341, 352, 333, and 328 ms, respectively; mean RTs for keys 1 to 5 for non-musicians were 759, 478, 503, 497, and 424 ms, respectively), *F*(1,22) = 3.99, *p* = .058, *η*_*p*_^*2*^ *=* .15. RTs changed as a function of Block, *F*(3,66) = 45.19, *ϵ* = .67, *p* < .001, *η*_*p*_^*2*^ *=* .67. Contrast analyses revealed a linear reduction in RT across blocks, *F*(1,22) = 59.59, *p* < .001, but also a quadratic trend, *F*(1,22) = 13.53, *p* = .001. These results indicate a general decrease in RT during learning, while the quadratic trend seems to indicate that this decrease was more pronounced in the initial learning phase. No interaction between Block and Group was observed, *p* = .17. However, trend analyses revealed a quadratic trend: *F*(1,22) = 6.56, *p* = .018, suggesting a stronger initial decrease of RTs for musicians than for non-musicians (see Fig 2). A main effect of Key was observed, *F*(4,88) = 129.83, *ϵ* = .37, *p* < .001, *η*_*p*_^*2*^ *=* .86, and an interaction between Key and Group was observed, *F*(4,88) = 8.20, *p* = .003, *η*_*p*_^*2*^ *=* .72. No group differences were found for the first button press (*p* = .56), which reflects the initiation time, but the subsequent keys characterizing the response sequence were pressed faster by musicians than by non-musicians (*p* < .04). No significant interaction between Block and Key, *F*(12,26) = 0.38, *ϵ* = .26, *p* = .78, *η*_*p*_^*2*^ *=* .02, and no significant interaction between Block, Key and Group was observed, *F*(12,26) = .87, *p* = .46, *η*_*p*_^*2*^ *=* .04.

**Fig 3:**
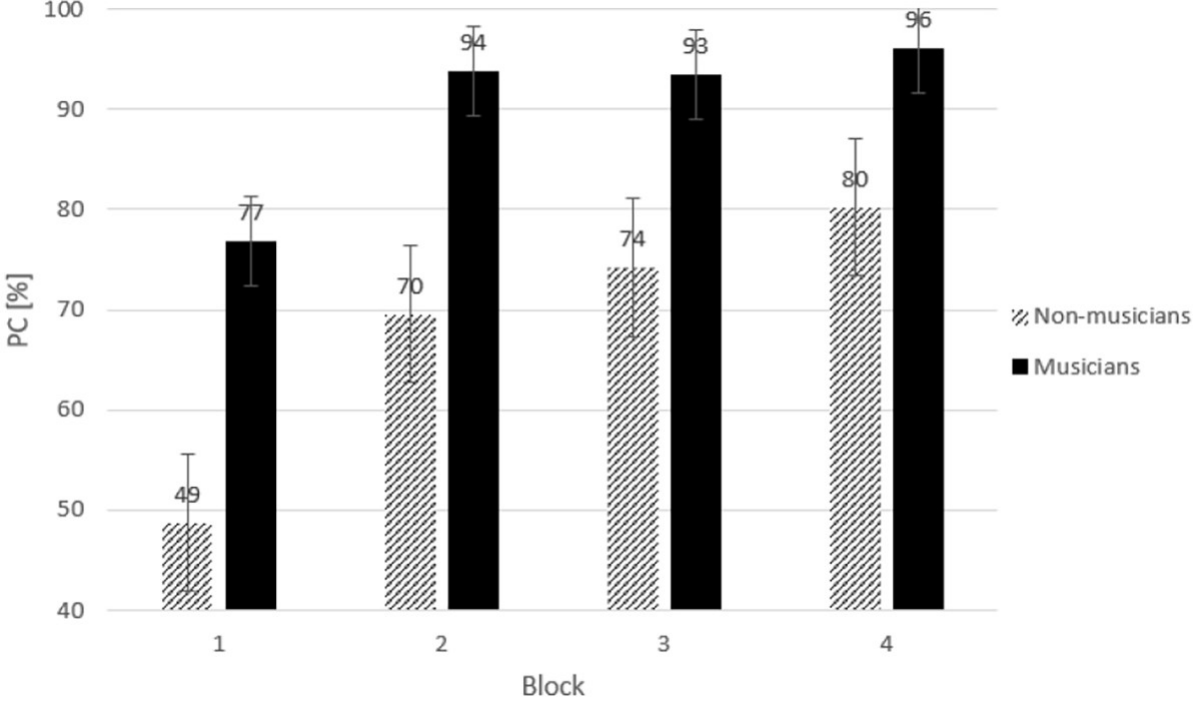
Correct response (PC) in percentages (%) of the total amount of to be executed sequences in the practice phase for each group. *Error bars* represent standard errors.

A repeated measures ANOVA was performed on arcsin transformed error percentages as a function of Group (2) and Block (4). A significant difference in accuracy was observed between musicians and non-musicians, *F*(1,22) = 11.89, *p* = .002, *η*_*p*_^*2*^ *=* .35. Musicians responded more accurately than non-musicians. A main effect of Block was observed, *F*(3,66) = 23.24, *ϵ* = .52, *p* < .001, *η*_*p*_^*2*^ *=* .51 (linear trend: *F*(1,22) = 29.51, *p* < .001; quadratic trend: *F*(1,22) = 13.0, *p* < .002; a cubic trend: *F*(1,22) = 10.1, *p* < .004). Inspection of Fig 3 suggests that the number of correct responses increased with practice and this effect was most pronounced in the early stage of learning. No significant interaction between Block and Group was observed, *F*(3,66) = 2.09, *p* = .15. These results indicate that the effect of practice on the number of correct responses was similar for musicians than for non-musicians (Fig 3).

#### The test phase

**Fig 4:**
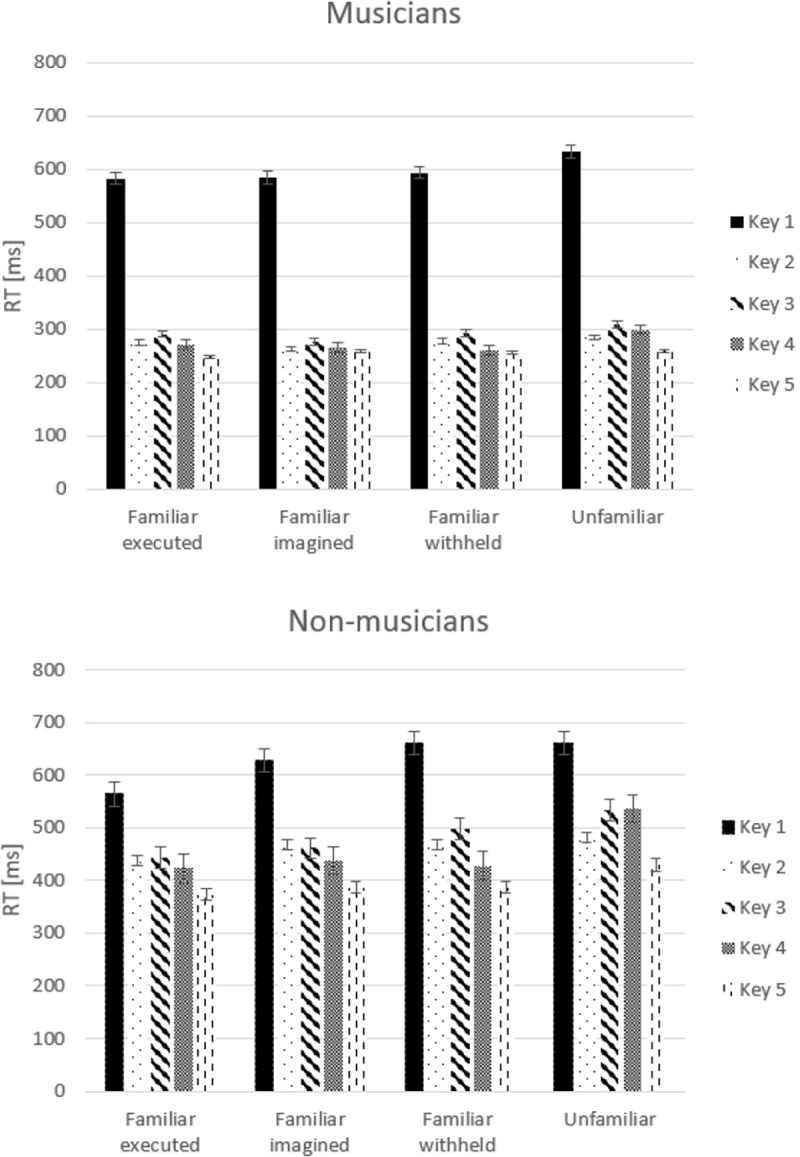
Mean response times (RTs) in milliseconds (ms) for each key presses (Key 1–5) as a function of Type of Sequence, for both groups separately. *Error bars* represent standard errors.

In the test phase, sequences that were executed before, imagined before, and withheld before now all had to be executed together with unfamiliar (not yet practiced) sequences to determine sequence-specific learning effects. Results showed significant differences in mean correct response time between the groups, *F*(1,22) = 13.29, *p* = .001, *η*_*p*_^*2*^ = .38. Musicians executed all the sequences faster than non-musicians. A significant difference as a function of Type of Sequence was observed, *F*(3,66) = 7.58, *ϵ* = .59, *p* = .002, *η*_*p*_^*2*^ = .26. Separate *t-*tests (one-tailed) revealed that: unfamiliar sequences were executed slower than familiar executed sequences, *t*(23) = 3.46, *p* = .001; unfamiliar sequences were executed slower than familiar imagined sequences, *t*(23) = 2.57, *p* < .01; and unfamiliar sequences were executed slower than familiar withheld sequences, *t*(23) = 1.75, *p* = .04. Results also revealed that familiar executed sequences were executed faster than familiar withheld sequences, *t*(23) = 1.7, *p* = .05; and familiar withheld sequences were executed slower than familiar imagined sequences, *t*(23) = 1.79, *p* = .04. No significant interaction between Type of Sequence and Group was observed, *F*(3,66) = 2.22, *p* = .13, *η*_*p*_^*2*^ = .09. A main effect of Key, *F*(4,88) = 60.33, *ϵ* = .25, *p* < 0.001, *η*_*p*_^*2*^ *=* .73, and an interaction between Key and Group was observed, *F*(4,88) = 5.38, *p* = .02, *η*_*p*_^*2*^ *=* .2, (a quadratic trend, *F*(1,22) = 7.32, *p* < .001). Results showed that the time to initiate the sequence was similar in both groups, but the time needed to execute the sequences (keys 2–5) was faster for musicians than for non-musicians. No significant interaction between Type of Sequence and Key was observed, *F*(12,26) = 2.01, *ϵ* = .43, *p* = .08, *η*_*p*_^*2*^ *=* .08, (Fig 4).

Although only a weak trend to an interaction between Type of Sequence and Group was observed, we tested our predictions directly to examine whether training with motor execution and motor imagery was more beneficial for musicians as compared with non-musicians. A separate ANOVA was performed with the factors: Type of Sequence (2, familiar executed/unfamiliar), Key (5), and Group (2). Furthermore, a separate ANOVA was performed with the factors: Type of Sequence (2, familiar imagined/unfamiliar), Key (5), and Group (2).

**Fig 5:**
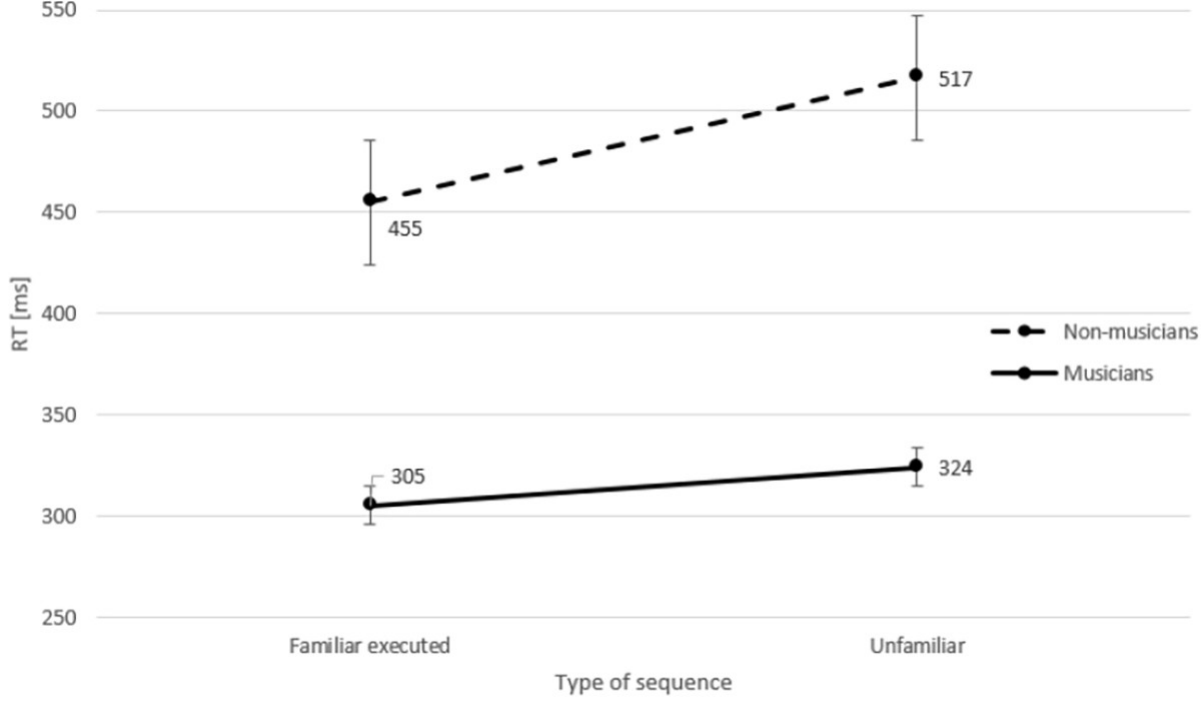
Mean response times (RTs) in milliseconds (ms) for familiar executed and unfamiliar sequences, for both groups separately. *Error bars* represent standard errors.

The results of training with motor execution again revealed faster execution of the sequences by musicians than by non-musicians, *F*(1, 22) = 11.21, *p* = .003, *η*_*p*_^*2*^ = .34. Furthermore, unfamiliar sequences were executed slower than familiar executed sequences, *F*(1, 22) = 26.6, *p* < .001, *η*_*p*_^*2*^ = .55. Most importantly, a significant interaction between Type of Sequence and Group was observed, *F*(1, 22) = 8.21,*p* = .009, *η*_*p*_^*2*^ = .22. Separate *t*-tests (one-tailed) were performed for each group. The results for musicians revealed that unfamiliar sequences were executed significantly slower than familiar executed sequences, *t*(11) = 3.1, *p* = .005; and for non-musicians the results also revealed that unfamiliar sequences were executed significantly slower than familiar executed sequences, *t*(11) = 2.92, *p* = .01. Inspection of Fig 5 shows a large difference in mean RTs between unfamiliar and familiar executed sequences for non-musicians (62 ms), while this difference was clearly much smaller for musicians (19 ms), which explains the observed interaction.

In the test phase, a similar repeated measures ANOVA was performed on arcsin transformed error percentages as a function of Group (2), and Type of Sequence (4). A significant difference in accuracy was observed between groups, *F*(1,22) = 20.06, *p* < .001, *η*_*p*_^*2*^ *=* .48. These findings show that musicians made less errors than non-musicians. A main effect of Type of Sequence was observed, *F*(3,66) = 10.69, *ϵ* = .57, *p* < .001, *η*_*p*_^*2*^ *=* .33.Separate *t-*tests revealed that the number of correct responses was significantly smaller for unfamiliar than for familiar executed sequences, *t*(23) = 3.89, *p* = .001, for unfamiliar sequences as compared to familiar imagined sequences, *t*(23) = 3.79, *p* = .001, and for unfamiliar sequences as compared to familiar withheld sequences, *t*(23) = 3.0, *p* = .006. No other significant differences in PC between sequences were observed, *p* > 0.8. No significant interaction between Type of Sequence and Group was observed, *p =* .12.

Similar as for RTs, to examine directly whether training with motor execution and motor imagery was more beneficial for musicians compared with non-musicians, first a separate ANOVA was performed with the factors: Type of Sequence (2, familiar executed/unfamiliar), and Group (2). Secondly, a separate ANOVA was performed with the factors: Type of Sequence (2, familiar imagined/unfamiliar), and Group (2).

The analysis for familiar executed and unfamiliar sequences revealed that musicians were more accurate than non-musicians, *F*(1,22) = 16.26, *p =* .001, *η*_*p*_^*2*^ *=* .43. A significant difference was observed as a function of Type of Sequence, *F*(1,22) = 19.06, *p* < .001, *η*_*p*_^*2*^ *=* .46, indicating that participants made more errors for unfamiliar sequences than for familiar executed sequences. No significant interaction between Type of Sequence and Group was observed, *F*(1, 22) = 3.5, *p* = .08, *η*_*p*_^*2*^ = .14.

The analysis for familiar imagined and unfamiliar sequences also revealed that musicians were more accurate than non-musicians, *F*(1,22) = 17.87, *p <* .001, *η*_*p*_^*2*^ *=* .45. A significant difference was observed as a function of Type of Sequence, *F*(1,22) = 17.73, *p* < .001, *η*_*p*_^*2*^ *=* .45, indicating that participants made more errors for unfamiliar sequences than for familiar imagined sequences. No significant interaction between Type of Sequence and Group was observed, *F*(1, 22) = 2.72, *p* = .11, *η*_*p*_^*2*^ = .11.

**Fig 6:**
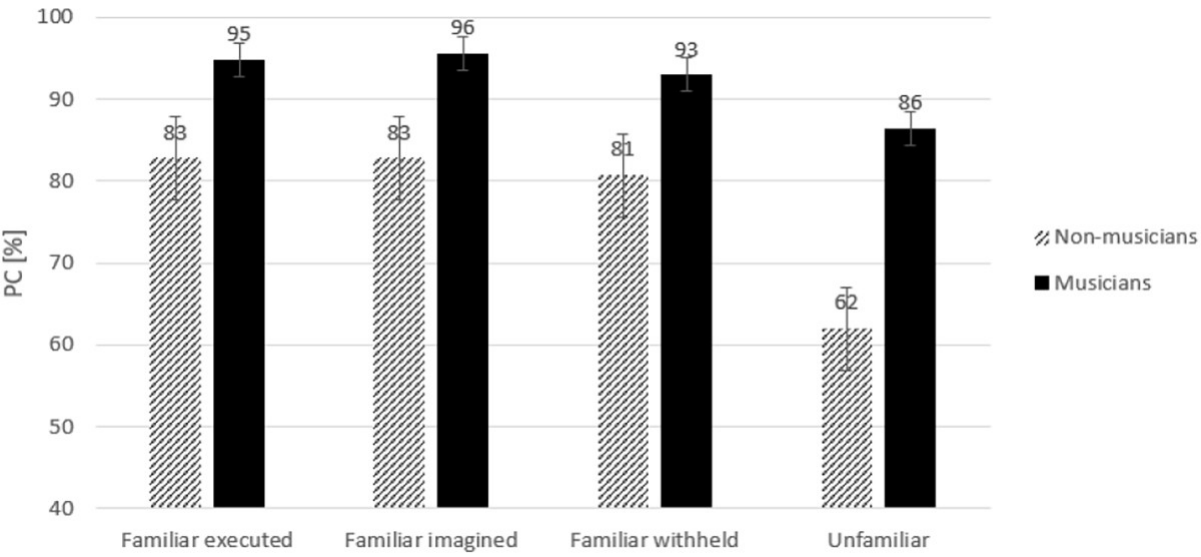
Percentage of correct responses (PC) in percentages (%) in the test phase for each type of sequence. *Error bars* represent standard errors.

Although no interaction between Type of Sequence and Group was observed, to examine whether musicians benefit more from motor execution and motor imagery than non-musicians during learning a motor skill, separate *t*-tests were performed for each group. In both groups, the number of correct responses was significantly smaller in the case of unfamiliar sequences as compared with familiar executed, familiar imagined, and familiar withhold sequences, *t*(11) > 2.48, *p* < .03. Inspection of Fig 6 clearly reveals that the highest number of errors was observed for unfamiliar sequences.

### EMG

**Fig 7:**
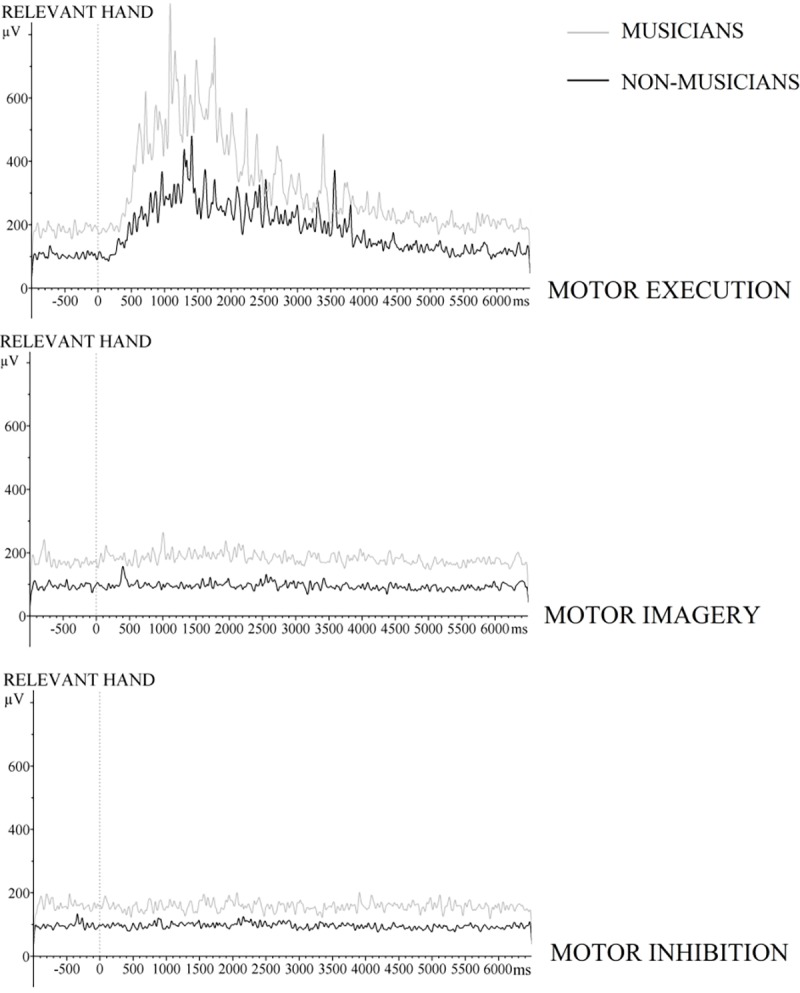
**Outcome of the wavelet analysis performed on the raw EMG signal measured from electrodes attached to the *left* and the *right* forearm**. The grand averages are only presented for the relevant hands for the motor execution, motor imagery, and motor inhibition from -1000 ms before the Go/NoGo signal (0 ms) to 6500 ms.

In the practice phase, EMG was measured to control whether participants did not flex their muscles in the case of motor imagery and motor inhibition as only during motor execution EMG activity should be observed. Fig 7 shows the averaged EMG signal for both groups while carrying out the required motor task (execution, imagery, or inhibition). No significant differences were observed between groups, *F*(1,22) = .05, *p* = 0.83, *η*_*p*_^*2*^ *=* .002. Results revealed a trend to a significant interaction between EMG-channel and Group, *F*(1,22) = 3.98, *p* = .06, *η*_*p*_^*2*^ *=* .15, These results suggest that muscular activity tended to be larger for the executing (relevant) hand for musicians than non-musicians. A main effect of Block was observed, *F*(3,66) = 6.09, *ϵ* = .72, *p* = .004, *η*_*p*_^*2*^ *=* .22, (a linear trend: *F*(1,22) = 9.07, *p* = .006; a quadratic trend: *F*(1,22) = 5.7, *p* = .026). A significant difference was observed as a function of Type of Sequence, *F*(2,44) = 115.03, *ϵ* = .55, *p* < .001, *η*_*p*_^*2*^ *=* .84. An inspection of Fig 7 shows higher muscular activity in the motor execution condition than in the motor imagery and motor inhibition conditions.

We were especially interested whether muscular activity differed between motor imagery and motor inhibition to determine whether participants possibly flexed their muscles in the case of motor imagery. Results revealed no significant difference as function of Type of Sequence, *p =* .11. Although participants did not move their fingers, some EMG activity seems present during motor imagery and motor inhibition.

### ERL results

Behavioral results from the practice phase revealed that the average time needed to execute the sequence was shorter for musicians than non-musicians, and sequences were additionally executed more accurately by musicians than by non-musicians. We questioned to what extent differences in behavioral performance between musicians and non-musicians are related to differences in ERLs, which were determined for each type of task (i.e., motor execution, motor imagery, and motor inhibition).

**Fig 8:**
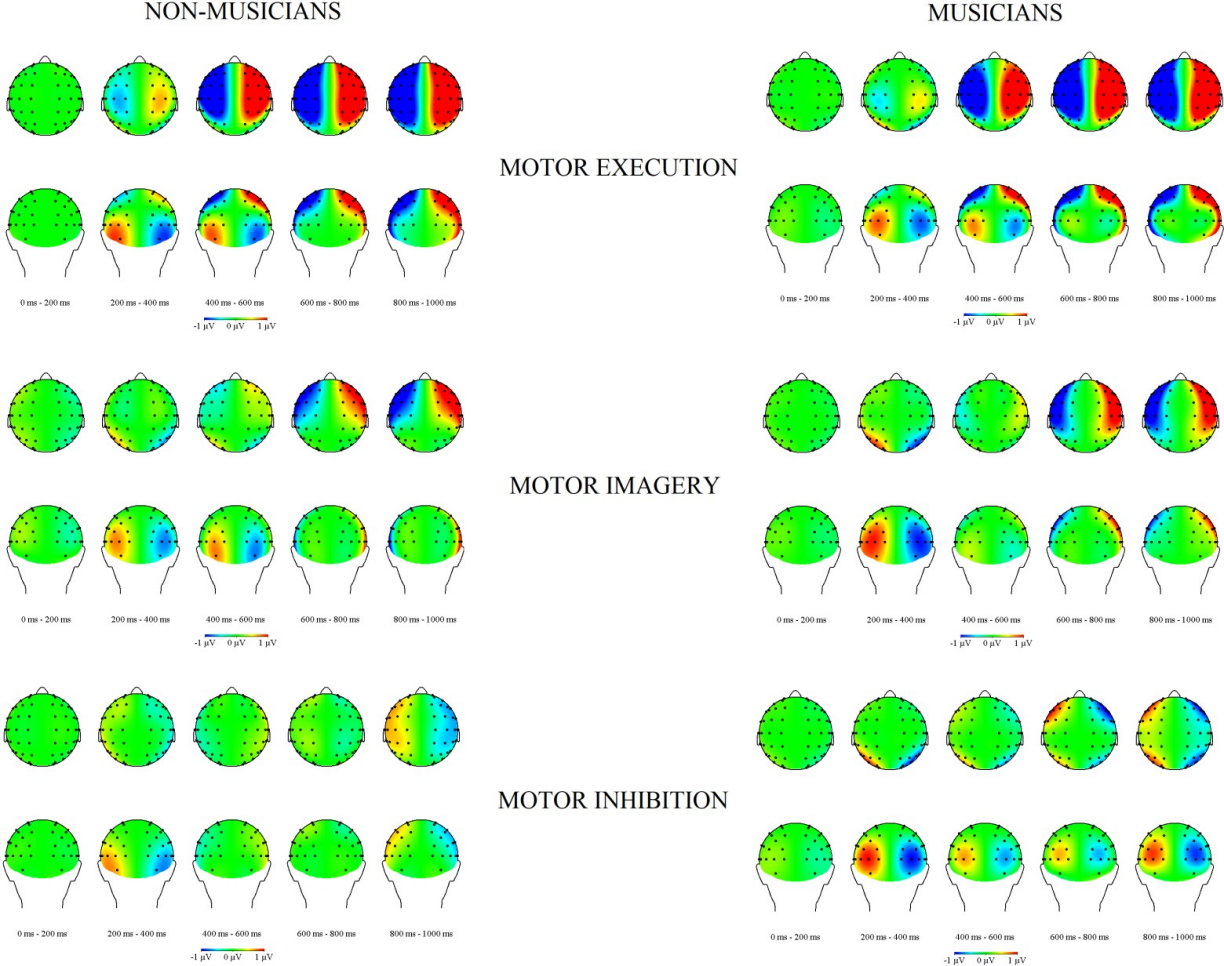
Topography of event related lateralizations (ERLs) for musicians and non-musicians for the three conditions (motor execution, motor imagery and motor inhibition) in the practice phase from the Go/NoGo signal (0 ms) to 1000 ms after the Go/NoGo signal. The left side of the brain displays the contra-ipsilateral difference. Negativity on the left hemisphere implies that activity was more negative on contralateral than on ipsilateral electrodes, which is visible for motor execution and motor imagery, while the opposite pattern seems to be present for motor inhibition.

Topographical maps for activity during the practice phase, from the onset of the Go/NoGo signal until 1000 ms are displayed in Fig 8 for each condition. ANOVAs were performed with the factors Group (2), and Type of Sequence (3), for each of two electrode pairs (C3/4, and CP3/CP4) that were selected on the basis of earlier results [7].

#### C3/C4 electrode pair

Separate analyses for lateralized activity (for two consecutive time windows) revealed a significant deviation from zero for motor execution starting from 280 ms to 1000 ms at C3/C4, being most pronounced from 680 ms to 720 ms, *t*(23) = -7.51, *p* < 0.001. For motor imagery, lateralized activity was observed starting from 480 ms to 880 ms at C3/C4, being most pronounced from 760 ms to 800 ms, *t*(23) = -3.48, *p* = .002. This increased negativity on the contralateral site as compared to the ipsilateral site in the case of motor execution and motor imagery is thought to reflect motor-related activity. For motor inhibition, a significant deviation from zero was only observed from 880 ms to 1000 ms, being most pronounced from 960 ms to 1000 ms, *t*(23) = 3.17, *p* = 0.004, however this activity concerned increased contralateral positivity.

No significant difference was observed between groups from the Go/NoGo signal (0ms) to 1000 ms, *p* > .2.The analysis with the factor of Type of Sequence revealed that ERLs differed from 240 ms to 1000 ms, being most pronounced from 800 to 840 ms, *F*(2,44) = 42.59, *ϵ* = .9, *p* < .001, *η*_*p*_^*2*^ *=* .66. Separate *t*-tests revealed that from 240 ms to 320 ms lateralized activity was significantly different only between motor execution and motor inhibition, *t*(23) > -3.19, *p* < .01. From 320 ms to 560 ms, more negativity on contralateral than on ipsilateral electrodes was observed in favor of motor execution compared with motor inhibition, *t*(23) > -6.72, *p* < .001; the analysis also revealed a significant difference between motor execution and motor imagery, revealing more negativity in the case of motor execution, *t*(23) > -5.5, *p* < .003. From 560 ms to 1000 ms, lateralized activity differed between all types of sequences. We observed a significant difference between motor execution and motor inhibition, *t*(23) > -10.79, *p* < .001, being most pronounced from 800 ms to 840 ms, *t*(23) = -10.79, *p* < .001. A significant difference was also observed between motor execution and motor imagery, *t*(23) > -4.4, *p* < .02, being most pronounced from 640 ms to 680 ms, *t*(23) > -4.4, *p* < .001. We also observed that motor inhibition differed from motor imagery, *t*(23) > 2.43, *p* < .02, being most pronounced from 800 ms to 840 ms, *t*(23) = 5.39, *p* < .001.These results demonstrated increased contralateral negativity for motor execution and motor imagery compared with motor inhibition. No significant interaction between Type of Sequence and Group was observed from 0 ms to 1000 ms, *p* > .07.

#### CP3/CP4 electrode pair

Separate analyses for lateralized activity at CP3/CP4 revealed a significant deviation from zero for motor execution starting from 280 ms to 1000 ms, being most pronounced from 480 ms to 520 ms, *t*(23) = -5.89, *p* < .001. For motor imagery, lateralized activity was observed starting from 160 ms to 240 ms, being most pronounced from 160 ms to 200 ms, *t*(23) = 2.9, *p* = .008. Similarly as at C3/C4 electrode pair, this increased negativity on the contralateral site as compared to the ipsilateral site is thought to reflect motor-related activity. For motor inhibition, no significant deviation from zero was observed at CP3/CP4.

No significant difference was observed between groups from 0 ms to 1000 ms, *p* > 0.1. The analysis with the factor of Type of Sequence revealed that ERLs differed from 280 ms to 1000 ms, being most pronounced from 680 to 720 ms, *F*(2, 44) = 14.75, *ϵ* = .9, *p* < .001, *η*_*p*_^*2*^ *=* .4. Separate *t*-tests revealed that from 280 ms to 680 ms lateralized activity was significantly different only between motor execution and motor inhibition, *t*(23) > -4.0, *p* < .001; and between motor execution and motor imagery, *t*(23) > -4.4, *p* < .001, showing more negativity in favor of motor execution. From 680 ms to 1000 ms, lateralized activity differed between all types of sequences. A significant difference was observed between motor execution and motor inhibition, *t*(23) > -4.89, *p* < .002, being most pronounced from 880 ms to 920 ms, *t*(23) = -4.89, *p* < .001. A significant difference was observed between motor execution and motor imagery, *t*(23) > -3.21, *p* < .03, being most pronounced from 640 ms to 680 ms, *t*(23) > -4.4, *p* < .001. We also observed that motor inhibition differed from motor imagery, *t*(23) > 2.43, *p* < .02, being most pronounced from 800 ms to 840 ms, *t*(23) = 5.39, *p* < .001.These results also demonstrated more increased contralateral negativity for motor execution and motor imagery as compared with motor inhibition. No significant interaction between Type of Sequence and Group was observed from 0 ms to 1000 ms, *p* > .44.

In conclusion, the results of our EEG analyses revealed similar lateralized activity for both groups while learning a motor skill, showing stronger contralateral activation of motor areas in the case of motor execution and motor imagery as compared with motor inhibition. The results revealed a polarity reversal in the case of motor inhibition above primary motor areas in both groups, which suggests a deactivation of motor areas.

## Discussion

This work aimed to investigate whether learning a fine sequential hand motor skill with motor execution and motor imagery is more efficient for musicians than for non-musicians. The experiment was divided into a practice phase (sequences had to be physically executed, imagined or inhibited), and a test phase in which all sequences had to be executed. Moreover, unfamiliar (i.e., unpracticed) sequences were added to the test phase to determine sequence-specific learning effects. As a consequence, we could establish whether motor learning is in general more efficient for musicians (a-specific learning effects), and whether sequence-specific learning effects might be influenced by increased expertise. Moreover, we were also interested whether this increased expertise of musicians may be reflected in electrophysiological changes in brain activation above cortical motor areas while imagining, inhibiting, and executing a required movement. First, we focused on learning a sequential motor skill with motor execution and possible group differences. Secondly, we examined the learning effects of motor imagery and possible group differences. Finally, we answered the question of whether increased motor expertise is reflected in electrophysiological changes in brain activation while executing, imaging, and inhibiting a fine motor skill between groups.

Considering the fact that musicians have to train fine motor skills significantly more often than novices, we hypothesized that learning a sequential motor skill with motor execution will differ between musicians and non-musicians. Behavioral results from the practice phase revealed a trend to an overall difference in mean RTs between musicians and non-musicians. Although the time required to initiate a sequence was similar for both groups, the averaged time needed to execute the rest of the sequence was shorter for musicians compared with non-musicians, suggesting that motor learning was more efficient for musicians. Moreover, sequences were executed more accurately by musicians. In both groups the number of correct responses increased with practice, and this effect was most pronounced in the early stage of learning, in line with our previous findings 7,41]. In conclusion, the results from the practice phase revealed that musicians learned response sequences more easily than non-musicians as their responses were faster and more accurate. Better motor performance in the practice phase for musicians may be related with the presence of enhanced visual cognition and improved sensorimotor integration, in line with previous studies [44, 45, 46, 47, 48, 49]. Moreover, several of those studies also pointed out that musicians are characterized by a greater efficiency of mnemonic processes [46, 47, 48, 49], which leads to better performance for musicians.

The better performance of musicians was evident in the test phase. Musicians executed all sequences (i.e., familiar executed, familiar imagined, familiar inhibited, and unfamiliar) faster and more accurately than non-musicians, indicating better sequence-a-specific learning effects for musicians. Moreover, by comparing unfamiliar sequences with familiar executed, familiar imagined, and familiar inhibited sequences in the test phase, we could also assess the influence of increased expertise on sequence-specific learning effects. Our results revealed that the difference in mean RTs between familiar executed sequences and unfamiliar sequences was actually greater for non-musicians than for musicians, which indicates sequence-specific learning was greater for non-musicians than for musicians. These results suggest that physical execution while learning the required motor sequence was more beneficial for non-musicians relative to musicians. In other words, a-specific learning effects for musicians were greater than sequence-specific learning effects.

Based on the notion that professional musicians regularly use motor imagery to improve their motor performance, we also questioned whether learning a motor skill with motor imagery is more beneficial for musicians than non-musicians. Similarly as for motor execution, motor imagery also induced sequence-specific learning effects, but these effects were independent of the increased expertise (regarding RTs and accuracy). These results suggest a comparable reinforcement of the structure of the motor sequence at a cognitive level for both non-musicians and musicians in the case of motor imagery. A possible reason why learning with motor imagery was beneficial for both groups is the lack of proprioceptive feedback during motor imagery in both groups. Proprioceptive feedback allows to regulate the proper pattern of muscle activation during movement which can be crucial to execute a required sequence movement accurate (see [41]). In contrast to the learning with motor execution, which was shown to be dependent on increased expertise, our results suggest that learning with motor imagery is more related with the development of the spatio-temporal aspects of a movement (constituting a motor program of a movement [4]) being independent of increased expertise.

As we demonstrated, musicians learned a motor skill more easily than non-musicians. In order to better understand how a motor skill was acquired by these two groups, we also examined whether increased motor expertise is reflected in electrophysiological changes in brain activation while executing, imaging, and inhibiting a fine motor skill. This was examined in the practice phase. We wanted to establish whether activation above motor areas (which are involved in motor execution and motor imagery) differs between musicians and non-musicians while learning a motor skill. In both groups, we observed increased negative lateralized activity above motor areas in the case of motor execution and motor imagery. In contrast to the findings of Baumann et al. (2007), we did not reveal a significant difference between the groups. Their study showed that sensorimotor regions in the human brain are more involved while imagining the motor movements associated with music performance for musicians than non-musicians [16]. Interestingly, previous research also indicated the increased activation of specific brain areas involved in the execution of the task (e.g., the primary motor cortex, the primary somatosensory cortex, the supplementary motor area), [15, 25]. The difference between our results and the aforementioned studies [15, 25, 16] could be due to different measurement methods (i.e., EEG and functional magnetic resonance imaging, respectively). Moreover, the computation of ERPs and ERLs implies that activity that is not strongly time-locked to a relevant event will be canceled out (e.g., see [50]). The employment of time-frequency analyses on the EEG such as wavelet analyses might reveal group differences that are not visible in ERPs. This is something that may need to be explored in a follow-up paper.

Our results also allowed to advance our understanding of acquiring a motor sequence. By measuring EEG we could establish how a motor sequence is acquired with motor execution, motor imagery and motor inhibition, and thereby we could verify previous findings [7, 8, 9]. The negativity above contralateral motor areas was related with motor activation during motor execution and motor imagery. Furthermore, ERLs revealed a polarity reversal in the case of motor inhibition, showing positivity on the contralateral hemisphere. These findings are in line with our previous study [7], which focused on the resemblance between motor execution and motor imagery relative to motor inhibition. Our ERLs results from the current study confirm the similar activation of brain motor areas during motor imagery and motor execution; whereas the activity during motor inhibition indicates a deactivation of motor areas.

Although the main purpose of this study concerned possible group differences in the acquisition of a fine motor skill, we could also determine sequence-specific learning effects for all participants as unfamiliar sequences were included next to the familiar imagined, familiar executed and familiar withheld sequences in both groups. The results from both groups revealed that unfamiliar sequences were executed slower than familiar executed, familiar imagined sequences, and familiar inhibited sequences indicating indeed sequence-specific learning effects. Moreover, the highest number of errors was observed for unfamiliar sequences, which were not practiced before for both groups. Interestingly, we observed that familiar inhibited sequences were executed more accurately than unfamiliar sequences. This can be related with the fact that inhibited sequences required motor preparation in the practice phase, even when they were not physically or mentally practiced. This result is in accordance with our previous study [41], showing that motor preparation may be already sufficient for learning a fine motor skill. In our previous study, we employed two groups of participants, who were instructed to imagine the movement sequence (a motor imagery group) or to inhibit the movement sequence after a NoGo signal (a control group). Behavioral results showed that both groups improved their RT and accuracy regardless of the different instruction. Even though participants did not receive an explicit instruction to imagine the movement, they may have imagined the movement during the preparation intervals. As a consequence they could also mentally practice the sequences. In the current study, we also revealed that familiar inhibited sequences were executed slower than familiar imagined sequences, which suggest that learning by motor imagery has a stronger effect than just learning by motor preparation and then motor inhibition.

The purpose of the present study was to evaluate whether learning a fine sequential hand motor skill with motor execution and motor imagery is more efficient for musicians than for non-musicians. Although the research has reached its aims, the potential limitations of this study should be considered. First, it can be argued that the results may be partly due to fact that participants could mentally execute the sequences in a different way as they were requested (i.e., to use motor imagery). We cannot exclude the fact that they imagined a sequence of sounds [1,51] even if they were instructed to imagine the execution of a required sequence. However, our EEG results clearly showed activation above motor areas; therefore, we favor the conclusion that participants indeed used motor imagery. A second limitation concerns possible sex differences, which could influence our results. Testing mainly females makes the generalization of the finding difficult [52]. It should also be noticed that the group of musicians in our study consisted only of pianists, which could have driven the obtained results. The required task in our experiment resembles the specific sensorimotor representation of a piano keyboard. Thus, it may be the case that different types of musicians (e.g., guitar players, violin players, drummers) would produce different results. In the future, more effort may also be put into clarifying the specific effects of motor imagery training among professional musicians, e.g., by measuring the functional changes protracted by increased expertise.

In conclusion, our results indicated that learning a fine motor skill only depends on increased expertise in the case of learning with motor execution. We showed that learning a fine motor skill with motor execution was more efficient for non-musicians relative to musicians. In the case of learning with motor imagery, we revealed its independence of increased expertise, as we observed similar sequence-specific learning effects in both groups. Nevertheless, we established that motor learning was in general more efficient for musicians (indicating a-specific learning effects) compared with non-musicians. These results support the notion that learning a motor skill is easier for musicians than for non-musicians. A comparison of electrophysiological activation during learning a fine motor skill between professional musicians and non-musicians revealed similar lateralized activity in both groups. In other words, we demonstrated that music experience did not influence electrophysiological brain activation above motor brain areas during learning a fine motor skill.

## Appendix

Sequences of five key presses used in the experiment:

6 structures of the sequence, 4 versions each

1-**a**, 2-**s**, 3-**d**, 4-**f**

1-;, 2-**l**, 3-**k**, 4-**j**

**Structure 1**

Version_1: Left hand: **a s f d s** (12432) Right hand: **; l j k l** (12432)

Version_2: Left hand: **s d a f d** (23143 Right hand: (23143)

Version_3: Left hand: **d f s a f** (34214) Right hand: (34214)

Version_4: Left hand: **f a d s a** (41321) Right hand: (41321)

**Structure 2**

Version_1: Left hand: **a d f s d**    (13423) Right hand: (13423)

Version_2: Left hand:                     (24134) Right hand: (24134)

Version_3: Left hand:                     (31241) Right hand: (31241)

Version_4: Left hand:                     (42312) Right hand: (42312)

**Structure 3**

Version_1: Left hand: **a f s a d**    (14213) Right hand: (14213)

Version_2: Left hand:                     (21324) Right hand: (21324)

Version_3: Left hand:                     (32431) Right hand: (32431)

Version_4: Left hand:                     (43142 Right hand: (43142)

**Structure 4**

Version_1: Left hand: **a d s f a**    (13241) Right hand: (13241)

Version_2: Left hand:                     (24312) Right hand: (24312)

Version_3: Left hand:                     (31423) Right hand: (31423)

Version_4: Left hand:                     (42134) Right hand: (42134)

**Structure 5**

Version_1: Left hand: **a f d a s**    (14312) Right hand: (14312)

Version_2: Left hand:                     (21423) Right hand: (21423)

Version_3: Left hand:                     (32134) Right hand: (32134)

Version_4: Left hand:                     (43241) Right hand: (43241)

**Structure 6**

Version_1: Left hand: **a f d a s**    (21431) Right hand: (21431)

Version_2: Left hand:                     (32142) Right hand: (32142)

Version_3: Left hand:                     (43213) Right hand: (43213)

Version_4: Left hand:                     (14324) Right hand: (14324)

## Supporting information

S1 File Additional results on RT in the test phase. (PDF)

S2 File Additional results on PC in the test phase. (PDF)
