# Supplementary material for: Do musicians learn a fine sequential hand motor skill differently than non-musicians?
Source: PLoS One. 2018 Nov 21;13(11):e0207449. doi: 10.1371/journal.pone.0207449 (PMC6248955; doi:10.1371/journal.pone.0207449)
Supplement: S1 File — (PDF) [file pone.0207449.s001.pdf]

S1 File. Additional results on RT in the test phase.

The results of training with motor execution revealed a faster execution of the sequences by musicians than by non-musicians, $F(1, 22) = 11.21$, $p = .003$, $\eta_p^2 = .34$. Furthermore, unfamiliar sequences were executed slower than familiar executed sequences, $F(1, 22) = 26.6$, $p < .001$, $\eta_p^2 = .55$. Most importantly, a significant interaction between Type of Sequence and Group was observed, $F(1, 22) = 8.21$,$p = .009$, $\eta_p{}^2 = .22$. Separate *t*-tests (one-tailed) were performed for each group (detailed results are presented in Table 1). The results for musicians revealed that unfamiliar sequences were executed significantly slower than familiar executed sequences, $t(11) = 3.1$, $p = .005$; and for non-musicians the results also revealed that unfamiliar sequences were executed significantly slower than familiar executed sequences, $t(11) = 2.92$, $p = .01$. Inspection of Fig 5 shows a large difference in mean RTs between unfamiliar and familiar executed sequences for non-musicians (62 ms), while this difference was clearly much smaller for musicians (19 ms), which explains the observed interaction. Again a main effect of Key was observed, $F(4, 88) = 48.79$, $\epsilon = .05$, $p < 0.001$, $\eta_p^2 = .69$, and an interaction between Key and Group was observed, $F(4, 88) = 6.26$, $p = .01$, $\eta_p^2 = .22$, (a linear trend: $F(1, 22) = 5.82$, $p < .03$; a quadratic trend: $F(1, 22) = 7.94$, $p = .01$). No significant interaction between Type of Sequence and Key was observed, $F(4, 88) = 2.35$, $\epsilon = .73$, $p = .08$, $\eta_p^2 = .1$.

Table 1. Results of *t*-tests on RT for each group comparing different types of sequence (i.e., familiar imagined, familiar executed, familiar withheld, and unfamiliar). * $p < 0.05$ (one-tailed test).

	Musicians		Non-musicians	
Type of sequence	$t(11)$	p	$t(11)$	p
Unfamiliar – familiar executed	3.1	**0.005***	2.92	**0.005***
Unfamiliar – familiar withheld	1.55	0.08	1.48	0.09
Unfamiliar – familiar imagined	3.99	**0.001***	2.0	**0.04***
Familiar imagined – familiar executed	0.51	0.31	0.89	0.2

Familiar withheld – familiar executed	1.95	**0.04***	1.17	0.14
Familiar withheld – familiar imagined	1.59	0.07	1.08	0.15

Results for the comparison of familiar imagined and unfamiliar sequences again revealed faster responses for musicians than for non-musicians, $F(1,22) = 13.35$, $p = .001$, $\eta_p^2 = .38$. Unfamiliar sequences were executed slower than familiar imagined sequences, $F(1, 22) = 9.45$, $p = .006$, $\eta_p^2 = .3$.No significant interaction between Type of Sequence and Group was observed, $F(1, 22) = .91$, $p = .35$, $\eta_p^2 = .04$. These results show that training with motor imagery was not more beneficial for musicians compared with non-musicians. Again, a main effect of Key, $F(4,88) = 47.9$, $\epsilon = .39$, $p < 0.001$, $\eta_p^2 = .69$, and an interaction between Key and Group was observed, $F(4,88) = 4.74$, $p = .02$, $\eta_p^2 = .18$, (a quadratic trend, $F(1,22) = 6.85$, $p < .02$). No significant interaction between Type of Sequence and Key was observed, $F(4, 88) = 1.96$, $\epsilon = .82$, $p = .12$, $\eta_p^2 = .08$.
