# Supplementary material for: Do musicians learn a fine sequential hand motor skill differently than non-musicians?
Source: PLoS One. 2018 Nov 21;13(11):e0207449. doi: 10.1371/journal.pone.0207449 (PMC6248955; doi:10.1371/journal.pone.0207449)
Supplement: S2 File — (PDF) [file pone.0207449.s002.pdf]

S2 File. Additional results on PC in the test phase.

Separate *t*-tests were performed for each group - results are presented in Table 2 - to examine whether musicians benefit more from motor execution and motor imagery than non-musicians during learning a motor skill, In both groups, the number of correct responses was significantly smaller in the case of unfamiliar sequences as compared with familiar executed, familiar imagined, and familiar withhold sequences, $t(11) > 2.48$, $p < .03$.

Table 2. *t*-test results of PC for each group comparing different types of sequence (i.e., familiar imagined, familiar executed, familiar withheld, and unfamiliar). * $p < 0.05$

	Musicians		Non-musicians	
Type of sequence	$t(11)$	p	$t(11)$	p
Unfamiliar – familiar executed	3.18	**0.009***	3.39	**0.006***
Unfamiliar – familiar imagined	4.16	**0.002***	3.08	**0.01***
Unfamiliar – familiar withheld	2.48	**0.03***	2.56	**0.03***
Familiar imagined – familiar executed	0.36	0.73	0.00	1.0
Familiar withheld – familiar executed	0.75	0.47	0.45	0.66
Familiar withheld – familiar imagined	1.02	0.33	0.71	0.49
